# Texting to Recruit Unassisted Tobacco Users at a Large Safety Net Health System Into Quitline Service for a Medicaid Value-Based Care Program: Pragmatic Randomized Controlled Trial

**DOI:** 10.2196/83269

**Published:** 2026-02-27

**Authors:** Cindy V Valencia, Melanie S Dove, Karen Kim, Paul Giboney, Hal F Yee Jr, Carrie A Kirby, Christopher M Anderson, Shu-Hong Zhu, Elisa K Tong

**Affiliations:** 1 Center for Healthcare Policy Research, University of California, Davis, 4150 V Street, Suite 2400, Sacramento, CA, 95817, United States; 2 Department of Public Health Sciences, University of California, Davis, Sacramento, CA, United States; 3 Los Angeles County Department of Health Services, Los Angeles, CA, United States; 4 Moores Cancer Center, University of California, San Diego, La Jolla, CA, United States; 5 Department of Internal Medicine and Center for Healthcare Policy Research, University of California, Davis, 4150 V Street, Suite 2400, Sacramento, CA, 95817, United States, 1 916 734 7005

**Keywords:** quitline, tobacco cessation, Spanish, safety net, clinic, text

## Abstract

**Background:**

A growing body of research supports the efficacy of text messaging programs to help tobacco users quit, but texting as a strategy for recruiting tobacco users into other evidence-based cessation services, such as quitline coaching, is less well understood. Texting to offer treatment could increase use of cessation resources, an important consideration for health systems trying to improve their quality metric performance on tobacco assessment and counseling.

**Objective:**

The aim of this study is to compare the effects of text messages offering free quitline coaching or free nicotine patches on engagement with quitline services by patients identified in electronic health records as unassisted tobacco users.

**Methods:**

Participants (N=4171) were adult patients of Los Angeles County Department of Health Services who had had a clinical visit in the past 12 months, were willing to receive text messages, and were identified as unassisted tobacco users (ie, those with no documented advice or assistance to quit in the past 24 months). They included 3139 English speakers and 1032 Spanish speakers. Participants were stratified by language, then randomly assigned to one of two groups. Group 1 received a text offering free quitline coaching. Group 2 received a text offering free nicotine patches. The texts were sent in April and May 2022. Outcome measures were the proportions calling the quitline and receiving evidence-based cessation treatments. Data were analyzed in 2025.

**Results:**

Overall, 1.5% (61/4171) of participants called the quitline, including 1.3% (28/2086) in Group 1 and 1.6% (33/2085) in Group 2, and 0.5% (21/4171) received treatment, including 0.4% (8/2086) in Group 1 and 0.6% (13/2085) in Group 2. There was no significant difference either in calls (*P*=.52) or receiving treatment (*P*=.29). However, Spanish speakers were significantly more likely to call than English speakers, 2.3% (24/1032) vs 1.2% (37/3139), respectively (*P*=.008), and engaged in treatment at approximately twice the rate of English speakers, 0.9% (9/1032) vs 0.4% (12/3139), although the latter difference was nonsignificant (*P*=.07).

**Conclusions:**

A single text was effective in connecting unassisted tobacco users with evidence-based quitline services for both English and Spanish speakers, but especially the latter. A population health approach of reaching out to tobacco-using patients outside clinical visits can meaningfully supplement provider referral. Research is needed on ways to increase the population health impact of this strategy.

## Introduction

The US Preventive Services Task Force urges health systems to support clinicians in treating tobacco use and calls on them to promote quitline utilization [1]. Quitlines are widely available and provide evidence-based cessation counseling [2]. Most partner with health systems to encourage clinicians to ask their patients if they use tobacco, advise those who do to quit, and connect them to quitlines via electronic referral (e-referral) [3,4]. Some quitlines provide additional services, including nicotine replacement therapy (NRT) and mobile health (mHealth) interventions [5].

mHealth interventions such as mobile apps and text messaging programs have received increasing attention due to their potential to expand the reach of tobacco cessation services. The approach is promising, given how widespread cell phone ownership is. In the United States, 98% of adults have a cell phone, including the same high percentage of White, Black, Latino, and English-speaking Asian adults [6]. In 2024, Americans exchanged nearly 2.2 trillion text messages [7]. Moreover, there is evidence that texting can improve the use of health care services. For example, sending appointment reminders via text increases attendance [8]. Text messages offering cessation treatment could potentially increase use of such services, an important consideration for health systems trying to improve their quality metric performance on tobacco assessment and counseling [9].

While there is growing evidence that text messaging programs can help tobacco users quit [10-12], less research has been done on texting as a way to recruit tobacco users into other evidence-based cessation services [13,14]. Researchers in the United Kingdom found that calls plus texts drove more recruitment for a text messaging trial than calls alone [15] and that sending a reminder about the trial plus a text drove more recruitment than the reminder alone [16]. A New York study reported that multimodal outreach including interactive voice response calls, emails, postcards, and texts led to a 6-fold increase in quitline reengagement [17]. Another New York study found that sending smokers identified in the electronic health record (EHR) 5‐7 texts resulted in 10.0% accepting quitline referral [13]. Researchers in Washington, DC, found that sending emergency department patients screened for tobacco use and willingness to receive up to 4 texts promoting a cessation program led 63.8% to opt in [14]. Illinois researchers reported that sending safety net health center patients identified as smokers in the EHR a letter plus 5 texts outperformed e-referral in driving quitline utilization [18]. Researchers in Vietnam found that frequent texting significantly increased quitline utilization [19]. All of these studies involved multiple text messages, additional modalities, or both.

This study, a pragmatic randomized control trial, implemented a text message campaign in a safety net health system promoting cessation in patients identified as unassisted tobacco users in the EHR. We compared two treatment offers and their uptake by English- and Spanish-speaking patients. The hypotheses were that texting would be a feasible means of recruiting unassisted tobacco users into quitline services, that offering free NRT would attract more enrollees than offering free coaching, and that recruiting by text would work equally well for English and Spanish speakers.

## Methods

### Study Participants and Intervention

Participants were patients of the Los Angeles County Department of Health Services (LADHS), the second largest municipal health system in the United States [20]. LADHS serves about 750,000 unique patients annually [20] and participates in a state Medicaid value-based care program to improve quality metric performance, including on “Tobacco Assessment and Counseling” [9]. Eligible patients were identified by the LADHS population health team using data extraction from the EHR for quality metric reporting. Eligibility criteria were being ≥18 years old, having had a clinical visit in the past 12 months, being willing to receive text messages, and being an unassisted tobacco user (ie, one with no documented advice or assistance to quit in the past 24 months). Of 4171 patients meeting these criteria, 3139 were English speakers and 1032 were Spanish speakers.

Participants were stratified by language, then randomly assigned to one of two groups. Randomization blocks were generated by the quitline, Kick It California, while participants were assigned and text messages were sent by LADHS. The messages were in English or Spanish according to the participant’s language preference. English speakers in Group 1 received a text reading, “Hello [first name], Kick It California works with your provider to help you quit smoking at no cost. A Quit Coach can double your chances of quitting. For more info call [toll-free phone number].” English speakers in Group 2 received a text reading, “Hello [first name], Kick It California works with your provider to help you quit smoking at no cost. You may be able to get nicotine patches mailed to your home at no cost. For more info call [toll-free phone number].” Spanish speakers in each group received a translated version of the message for that group. Four dedicated toll-free phone numbers were used, each of which was only available to members of a single group who spoke either English or Spanish. These steps were taken to enable tracking of incoming calls and to provide callers with linguistically appropriate services. The texts were sent in April and May 2022.

Staff of the quitline who handled incoming calls were blind to the allocation of participants and offered coaching to all who enrolled. Coaching followed a structured, multicall protocol previously proven effective [21,22] and described elsewhere [23]. Quitline staff also offered a free 2-week supply of nicotine patches to all who enrolled unless NRT was contraindicated, regardless of which text message they received. The patches were mailed directly to participants’ homes. To ensure equitable access, a second text message offering free patches was sent to all participants in June 2022.

### Measures

Demographic information obtained from the EHR included age, sex, race/ethnicity, and language. The outcome measures were the proportions of participants calling the quitline and receiving evidence-based cessation treatments, which could include NRT, quitline coaching, or both. Calls to the quitline and treatments received were attributed to the initial text message only if they took place prior to when the second text message was sent. For those who received coaching, the number of follow-up sessions was also tracked.

### Analysis

Descriptive statistics were used to describe patient characteristics by treatment condition. The proportions of participants calling the quitline and receiving treatment (NRT, coaching, or both) were calculated for each treatment and language group. Differences between treatment groups were assessed using *χ*^2^ tests for categorical variables or the Fisher exact test in the event of small cell sizes. Analyses were conducted in 2025 using SAS (version 9.4; SAS Institute Inc).

### Ethical Considerations

The study was approved as exempt from human subjects review by the University of California Davis institutional review board (application number 1301241‐6) and the LADHS institutional review board (application number 2018-12-785). Because the institutional review board determination was that the study was not research, informed consent was not required. Data were deidentified to safeguard participant information. Participants were not offered compensation. The trial was registered on ClinicalTrials.gov (NCT07102290).

## Results

Table 1 shows the demographic characteristics, by treatment group, of LADHS patients identified as unassisted tobacco users in the EHR who were randomized into the study. There were no significant differences between groups with regard to age, sex, race/ethnicity, or language, suggesting that the groups were equivalent at baseline.

**Table 1.** Characteristics of text message recipients in the Los Angeles County Department of Health Services system by randomized condition (N=4171).

	Group 1, free coaching (n=2086), %	Group 2, free nicotine replacement therapy (n=2085), %	*P* value^a^
Age (years)			
18-24	2.2	2.3	.49
25-44	31.5	30.4	
45-64	52.9	52.2	
>64	13.5	15.1	
Sex			
Male	63.1	60.4	.13
Female	36.9	39.6	
Other	0.05	0.05	
Race/ethnicity			
American Indian/Alaska Native	0.34	0.58	.25
Asian	2.5	3.0	
Black	25.9	25.1	
White	6.5	7.1	
Hispanic/Latino	43.9	44.1	
Non-Hispanic other^b^	19.3	17.8	
Unknown/blank/refused	1.6	2.4	
Language			
English	75.3	75.3	.99
Spanish	24.7	24.8	

a *P* value from *χ*2 test for all variables except sex, which used the Fisher exact test because of cell sizes <5.

b Non-Hispanic other includes Pacific Islander, other race, and multiple races.

**Figure 1.**
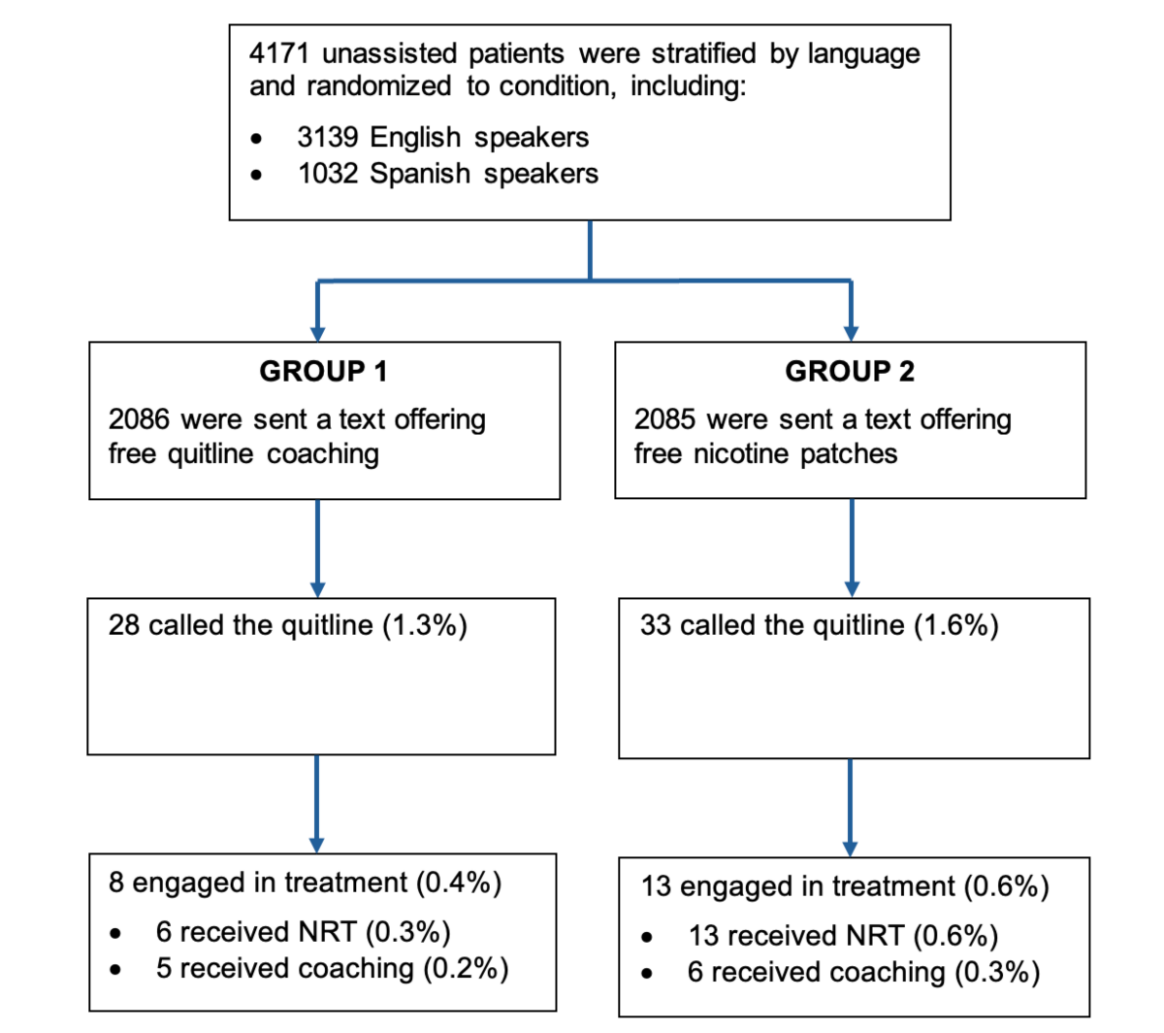
Flow of Los Angeles County Department of Health Services text message recipients through the study.

Figure 1 summarizes the flow of participants through the study. Of 4171 LADHS patients identified in the EHR as unassisted tobacco users, half (n=2086) were randomly assigned to Group 1 and received a text message offering free quitline coaching. The other half (n=2085) were randomly assigned to Group 2 and received a text message offering free NRT. A total of 61 participants responded to the text they received by calling the quitline, including 28 in Group 1 (1.1%) and 33 in Group 2 (1.6%). Of these, 21 participants engaged in treatment, including 8 in Group 1 (0.4%) and 13 in Group 2 (0.6%). Most of these (n=19) received NRT, including 6 in Group 1 (0.3%) and 13 in Group 2 (0.6%), while 11 received a coaching session to help them prepare to quit, including 5 in Group 1 (0.2%) and 6 in Group 2 (0.3%). Eight participants also completed at least one follow-up session to help them stay on track with quitting, completing 2.1 follow-up sessions on average (not shown in figure).

Table 2 shows in more detail the effects of the intervention on calls to the quitline by treatment condition and language. There was no significant difference by treatment condition, with 1.3% (28/2086) of Group 1 participants and 1.6% (33/2085) of Group 2 participants calling the quitline (*P*=.52), nor were there between-group differences within languages. Among English speakers, 1.1% (17/1570) in Group 1 called the quitline vs 1.3% (20/1569) in Group 2 (*P*=.62), and among Spanish speakers, 2.1% (11/516) in Group 1 called the quitline vs 2.5% (13/516) in Group 2 (*P*=.68). However, when examined by the stratification variable, language, there was a significant difference, with Spanish speakers twice as likely as English speakers to call the quitline, 2.3% (24/1032) versus 1.2% (37/3139), respectively (*P*=.008).

**Table 2.** Calls to the quitline by Los Angeles County Department of Health Services text message recipients by randomized condition and language.

Language	Group 1, free coaching (n=2086), % (n/N)	Group 2, free nicotine replacement therapy (n=2085), % (n/N)	*P* value^a^	Total (N=4171), % (n/N)	*P* value^b^
English	1.1 (17/1570)	1.3 (20/1569)	.62	1.2 (37/3139)	.008
Spanish	2.1 (11/516)	2.5 (13/516)	.68	2.3 (24/1032)	
Total	1.3 (28/2086)	1.6 (33/2085)	.52	1.5 (61/4171)	

a *P* value from *χ*2 test comparing Group 1 to Group 2 for each language category.

b *P* value from *χ*2 test comparing English to Spanish.

Table 3 shows the effects of the intervention on treatments received. Overall, 21 of 4171 or 0.5% of participants received evidence-based treatment, including 0.4% (8/2086) in Group 1 and 0.6% (13/2085) in Group 2. As with calls to the quitline, there was no significant difference by treatment condition in this outcome (*P*=.29), nor were there between-group differences within languages. Among English speakers, 0.3% (5/1570) in Group 1 engaged in treatment versus 0.4% (7/1569) in Group 2 (*P*=.58), and among Spanish speakers, 0.6% (3/516) in Group 1 engaged in treatment versus 1.2% (6/516) in Group 2 (*P*=.51). Spanish speakers engaged in treatment at approximately twice the rate of English speakers, but the difference was nonsignificant, 0.9% (9/1032) versus 0.4% (12/3139), respectively (*P*=.07).

**Table 3.** Los Angeles County Department of Health Services text message recipients’ receipt of tobacco cessation treatment by randomized condition and language.

Language	Group 1, free coaching (n=2086), % (n/N)	Group 2, free nicotine replacement therapy (n=2085), % (n/N)	*P* value^a^	Total (N=4171), % (n/N)	*P* value^b^
English	0.3 (5/1570)	0.4 (7/1569)	.58	0.4 (12/3139)	.07
Spanish	0.6 (3/516)	1.2 (6/516)	.51	0.9 (9/1032)	
Total	0.4 (8/2086)	0.6 (13/2085)	.29	0.5 (21/4171)	

a *P* value from Fisher exact *χ*2 test comparing Group 1 to Group 2 for each language category.

b *P* value from Fisher exact *χ*2 test comparing English to Spanish.

## Discussion

### Principal Findings

This pragmatic trial tested a strategy of sending unassisted tobacco users in a safety net health system a text message promoting quitline services: either free coaching or free NRT. Prior to the trial, clinicians in the system could e-refer patients to the quitline, but use of this mechanism tends to be low [24,25]. The trial offered an opportunity to test a population health strategy of reaching out to tobacco users outside clinical visits, targeting those who had not recently received help to quit. The intervention resulted in 1.5% of identified tobacco users calling the quitline and 0.5% receiving one or more evidence-based treatments. The latter rate is comparable to the treatment reach rate of US quitlines, which is less than 1% annually [5]. Moreover, it resulted from a low-intensity outreach effort—a single text.

The offers of free coaching and free NRT were equally effective at driving enrollment. This result was unexpected, as previous research found that mailers sent to Medicaid households were much more effective at driving quitline utilization when they offered free NRT than when they offered more generic help [26]. By targeting unassisted tobacco users, the texts may have reached an audience more concerned with obtaining any help to quit than a particular kind of help. The texts may also have done a better job “selling” the coaching than the NRT, as the coaching offer included the message, “a Quit Coach can double your chances of quitting.” The NRT offer, in contrast, made no explicit attempt to boost positive expectancy. In any case, there was no discernible difference in the two offers’ ability to motivate enrollment.

In contrast, there was a notable difference by language: Spanish speakers called the quitline and engaged in treatment at twice the rates of English speakers. These are encouraging results, given that Latino tobacco users are less likely to receive clinical advice to quit than non-Latino White tobacco users [27]. Latinos and Spanish speakers often lack knowledge about available cessation resources, hold misconceptions about cessation treatment, and may view tobacco use as a personal weakness, rather than as a condition to be treated [28-30]. Moreover, there is evidence that while Spanish speakers face barriers accessing digital health information, such as through patient portals, text messaging actually facilitates their access [31]. This may help explain why receiving a language-concordant text from their health system appears to have increased engagement with the quitline among Spanish-speaking patients. Regardless, the result suggests that population health strategies like the one tested in this study could help address disparities in the receipt of clinical advice.

The study adds to previous research on the use of texting to recruit patients into cessation treatment. Several studies have examined texting either alone [13,14,19] or as part of a multimodal outreach approach [15-18]. All studies in the former category involved multiple texts. Taken together, these studies have shown that texting can be effective in recruiting tobacco users into cessation service in the context of multitext or multimodal campaigns. This study adds to this body of research by confirming that recruiting tobacco users via text is feasible and showing that even a single text can increase recruitment.

The lesson we draw from this finding is not that organizations using multiple text messages to recruit tobacco users into evidence-based cessation treatment or using text messages in combination with other modalities should consider downgrading to a single-text format, but rather that this low-cost, almost universally available outreach tool is robust enough to measurably increase recruitment even with a single dose. It speaks to the value of quitlines partnering with health systems on text messaging campaigns to reach tobacco-using patients outside of clinical visits to offer help with quitting. Such population health strategies have the potential to meaningfully supplement direct health care provider treatment and referral [32] and to improve quality metric performance for safety net health systems in a state Medicaid value-based care program without additional staffing costs or burden [9]. Complementary strategies may further increase the effectiveness of text messaging as a recruitment tool, including using it to reach out to additional groups of patients, using opt-out approaches [33], increasing the number and variety of messages sent [13], using dynamic tailoring [34], offering incentives [26], and offering other mHealth services [14].

The public health significance of this low-cost approach is that other health systems can quickly and widely adopt it to improve population-based tobacco quality metrics, which would have the additive effect of increasing the treatment reach of evidence-based resources such as quitlines. Starting in 2026, community cancer centers accredited by the American College of Surgeons Commission on Cancer, which care for 70% of the nation’s patients with cancer, have a new standard to screen every patient newly diagnosed with cancer for smoking and to offer a referral within 30 days [35]. Also in 2026, health plans have a new population-based metric for “Tobacco Use Screening and Cessation Intervention” [36]. Low-cost proactive outreach strategies like the one described in this study can help cancer centers, health plans, provider networks, and other health care entities reach beyond clinical encounters to offer tobacco treatment to their members and patients who may need it.

To our knowledge, this study is the first to show that texting may be a more effective recruitment tool among Spanish-speaking tobacco users than among English-speaking tobacco users. This also speaks to the value of quitlines and health systems partnering to reach underserved patients [27]. All state quitlines in the United States serve both English and Spanish speakers; therefore, all health systems could potentially partner with their quitline on bilingual text messaging campaigns to reduce disparities in access to evidence-based cessation services [37].

### Limitations

This study had limitations. First, only unassisted tobacco users were included in the study; the response and engagement rates may have differed if assisted tobacco users were included. Second, there was no means to identify patients with incorrect phone numbers in the participant list, and there was no way to know whether text messages were received unless patients contacted the quitline. Third, it is unknown whether participants enrolling in the quitline benefited from the service, as the trial was not powered or designed to assess quitting outcomes. Fourth, the study included only one safety net health system, limiting generalizability. Finally, the trial was conducted a little more than two years into the COVID-19 pandemic, and it is unknown to what extent COVID-19–related stressors such as social, economic, and psychosocial distress may have affected patients’ willingness to respond to a text message offering help to quit.

### Conclusion

This study showed that a single text message can effectively connect unassisted tobacco users to evidence-based cessation services for both English and Spanish speakers, but especially the latter. It showed that a population health approach of reaching out to tobacco-using patients outside clinical visits can meaningfully supplement provider referral. This population health strategy is being disseminated to and adapted by other safety net health systems and state Medicaid managed care plans in a statewide learning collaborative [38,39]. Future research should examine ways of increasing the population health impact of the strategy.

## Supplementary material

10.2196/83269 Checklist 1 CONSORT checklist.

## Abbreviations

CONSORT: Consolidated Standards of Reporting Trials
e-referral: electronic referral
EHR: electronic health record
LADHS: Los Angeles County Department of Health Services
mHealth: mobile health
NRT: nicotine replacement therapy
